# Supplementary material for: Transcriptomic analysis of the lesser spotted catshark (Scyliorhinus canicula) pancreas, liver and brain reveals molecular level conservation of vertebrate pancreas function
Source: BMC Genomics. 2014 Dec 6;15:1074. doi: 10.1186/1471-2164-15-1074 (PMC4362833; doi:10.1186/1471-2164-15-1074)
Supplement: Supplementary file 2 — Additional file 2: Details of antibodies and absorption assays used in immunohistochemistry experiments. (PDF 97 KB) [file 12864_2014_6933_MOESM2_ESM.pdf]

Additional file 10.

Additional table 1. Table of antisera used in immunohistochemical surveys of the catshark pancreas. PYY, peptide YY; NPY, neuropeptide Y: PP, pancreatic polypeptide.

Antigen	Species	Dilution	Source
PYY	Rabbit	1:500	Sigma
PYY	Rabbit	1:500	AbCam, UK
NPY	Rabbit	1:1000	AbCam,UK
Insulin	Mouse	1:75	Novo Nordisk A/S, Denmark
Insulin	Guinea Pig	1:200	DAKO, Glostrup, Denmark
Glucagon	Mouse	1:50	Novo Nordisk A/S, Denmark
Glucagon	Rabbit	1:500	DAKO, Glostrup, Denmark
PP	Rabbit	1:500	Sigma
PP	Guinea Pig	1:500	Linco/Millipore
Somatostain	Mouse	1:100	Novo Nordisk A/S, Denmark

Additional table 2. Absorption of Pancreatic Polypeptide Family antisera. Staining is characterised from strong (+++) to weak (+) or absent (-). PYY, peptide YY; NPY, neuropeptide Y: PP, pancreatic polypeptide.

Antibody	Peptide	Staining Dogfish	Staining Mouse
PP	None	+++	-
PP	NPY	-	-
PP	PYY	-	-
PP	PP	-	-
PYY abcam	none	++	++
PYY abcam	PP	++	++
PYY abcam	NPY	+	++
PPY abcam	PYY	-	-
PYY Sigma	none	+++	+
PYY Sigma	PP	+++	+
PYY Sigma	NPY	+++	+
PYY sigma	PYY	-	-
NPY	none	+++	+++
NPY	PP	+++	+++
NPY	PYY	-	-
NPY	NPY	-	-
